# Weight Loss Patterns and Clinical Outcomes of GLP1 Receptor Agonists in Breast Cancer Survivors

**DOI:** 10.1158/2767-9764.CRC-25-0554

**Published:** 2026-03-02

**Authors:** Jasmine S. Sukumar, Akshara S. Raghavendra, Sarah Pasyar, Roland L. Bassett, Debu Tripathy, Carlos H. Barcenas, Karen M. Basen-Engquist, Banu K. Arun

**Affiliations:** 1 Division of Cancer Medicine, Department of Breast Medical Oncology, The University of Texas MD Anderson Cancer Center, Houston, Texas.; 2 Department of Biostatistics, The University of Texas MD Anderson Cancer Center, Houston, Texas.; 3 Department of Health Disparities Research, The University of Texas MD Anderson Cancer Center, Houston, Texas.

## Abstract

The use of glucagon-like peptide-1 receptor agonists (GLP1-RA) for weight loss is increasing; implications in breast cancer survivors remain unclear. This retrospective cohort study evaluated treatment patterns, weight loss, and outcomes in breast cancer survivors receiving GLP1-RA at an academic institution. We evaluated patients with nonmetastatic [ductal carcinoma *in situ* (DCIS), stage 1–3] breast cancer who received GLP1-RA (2005–2024). Linear regression models estimated associations between weight change and clinical factors. After excluding DCIS, propensity score matching (1:2) was used to match patients who received GLP1-RA with patients who did not, based on confounding covariates. Kaplan–Meier estimates and log-rank tests compared disease-free survival (DFS) and overall survival (OS) between GLP1-RA users versus nonusers in the subgroup with invasive disease. We identified 1,022 patients; 79% had type 2 diabetes mellitus. The median weight and body mass index at GLP1-RA initiation were 86.8 kg (47.2–175 kg) and 33.5 kg/m^2^ (18.9–61.8 kg/m^2^). In semaglutide or tirzepatide users (442, 43.2%), the median weight change at 3, 6, and 12 months after GLP1-RA initiation was −1.9% (−13.2% to 14.9%), −3.1% (−20.2% to 19%), and −2.6% (−27.8% to 11.5%), respectively. Endocrine therapy and metformin use were associated with weight gain and loss, respectively; invasive disease stage was linked to greater weight loss. GLP1-RA was not associated with DFS, but OS significantly differed between GLP1-RA users (*n* = 810) and nonusers (*n* = 1,620; hazard ratio, 0.37; 95% confidence interval, 0.27–0.53; *P* < 0.0001). In conclusion, in this real-world study in breast cancer survivors, GLP1-RA was associated with modest weight loss and improved all-cause survival. Clinical trials are warranted to study GLP1-RA in this population.

**Significance:**

This is the largest study describing real-world patterns of GLP1 receptor agonists in breast cancer survivors. Clinical trials should evaluate these agents for weight management as an adjunct to lifestyle interventions and the potential role in cancer control in breast cancer survivors.

## Introduction

The number of breast cancer survivors continues to increase with advances in early detection and treatment (1). Type 2 diabetes mellitus (DM2) in breast cancer survivors is associated with increased symptom burden, treatment complications, and greater mortality (2). Moreover, obesity in breast cancer survivors is prevalent and linked with adverse health sequelae, including a higher risk of comorbidities (e.g., cardiovascular disease and DM2) and compromised quality of life (e.g., fatigue, sexual dysfunction, negative body image, lymphedema, and neuropathy; refs. 3, 4). Observational data support that obesity in breast cancer survivors is associated with increased recurrence risk and mortality, demonstrated across disease subtypes (hormone positive and negative) and independent of menopausal status (3–5). Weight gain following a breast cancer diagnosis is also common and a negative prognostic factor. Hence, targeting cardiometabolic risk and maintaining an ideal body weight is a key tenet of optimal health in breast cancer survivorship (6).

Glucagon-like peptide-1 receptor agonists (GLP1-RA) and dual GLP1-RA/glucose-dependent insulinotropic polypeptide receptor agonists (GIP-RA) are incretin-based drugs with favorable metabolic effects and are approved for DM2 and weight management in overweight/obesity (7–9). GLP1-RA and GIP-RA agents regulate postprandial glucose metabolism, promote satiety, and decrease gastric emptying. They have shown consistent benefit in glycemic control and reduction in energy intake, leading to significant weight loss (7–9). Their additional health benefits include reduced cardiovascular risk and improved all-cause mortality in certain populations, such as those with DM2 (9–12). The utilization of these agents is rapidly increasing, but their clinical implications in breast cancer survivors are not well understood. There have been few prior studies specifically in breast cancer populations, albeit with small sample sizes (13–15). This retrospective cohort study evaluated real-world patterns of GLP1-RA utilization in patients with breast cancer. We also explored the impact of these drugs on weight change, evaluating clinical factors associated with weight loss efficacy. Moreover, we investigated the relationship of GLP1-RA with breast cancer recurrence risk and mortality [disease-free survival (DFS) and overall survival (OS)].

## Materials and Methods

This retrospective cohort study was approved by The University of Texas MD Anderson Cancer Center (MDACC) institutional review board and followed Strengthening the Reporting of Observational Studies in Epidemiology (STROBE) guidelines.

### Population

**Figure 1.**
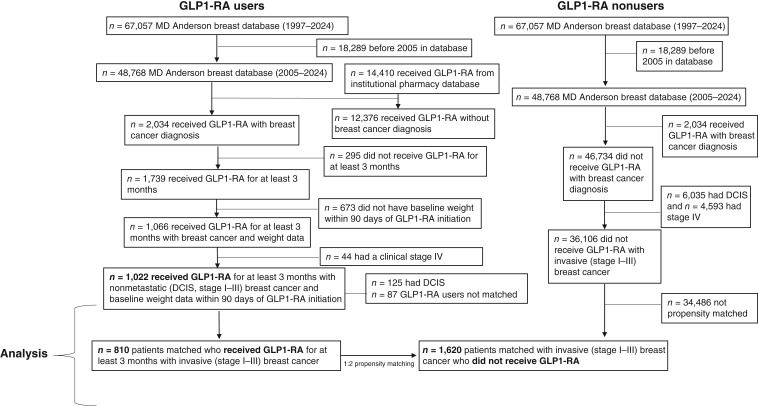
Patient selection flow chart.

We identified patients diagnosed with nonmetastatic breast cancer between January 2005 (the first year in which GLP1-RAs were approved for DM2) through January 2024 who were 18 years or older, had body weight data available (before and after GLP1-RA initiation), and received GLP1-RA for at least 3 months (Fig. 1). The MDACC pharmacy database serves as a complementary data source for medication exposure and captures detailed records of systemic anticancer and noncancer therapies dispensed. Medication records were comprehensive, incorporating drug names with doses and route of administration, reconciled appointment-based medication lists, prescriptions through patient-reported medication histories and admission/discharge summaries, documented home medications, and prescriptions dispensed within and outside MDACC (retail medications). GLP1-RA agents included exenatide, liraglutide, semaglutide, dulaglutide, lixisenatide, albiglutide, and the dual GLP1/GIP-RA tirzepatide.

Individuals identified from the pharmacy database without a breast cancer diagnosis were excluded. The resulting cohort of patients with breast cancer was then evaluated in the MDACC Breast Medical Oncology Management System (BCMS) database. This is a well-annotated prospectively gathered database of patients with breast cancer at the academic institution. Clinical data such as demographics, clinicopathologic features, and breast cancer treatment history were obtained from the electronic medical records (EMR) at MDACC, with linkage to the institutional Tumor Cancer Registry, which queries the Social Security Death Index and Texas Bureau of Vital Statistics and external databases for prescription and outcome verification.

### GLP1-RA data collection

The start date and duration of GLP1-RA treatment were determined from the medication list in the EMR, updated at each patient visit. The start date was defined as the earliest pharmacy dispensing date (first visit) for each patient. The 3-month treatment duration was defined as continuous medication supply for at least 90 days. Individuals who used more than one GLP1-RA for longer than 3 months (indicative of a switch) were classified according to the first GLP1-RA used.

### Clinical data collection

Nonmetastatic breast cancer diagnosis was defined as a diagnosis of ductal carcinoma in situ (DCIS) or stage I to III invasive breast cancer. Clinical staging was defined by the American Joint Committee on Cancer guidelines at diagnosis. The collected demographic and clinical data encompassed age, sex, menopausal status, DM2 (defined as a DM2 diagnosis and/or prior receipt of insulin), metformin and insulin use, body weight, body mass index (BMI), race/ethnicity, cancer stage, breast cancer subtype, (neo)adjuvant treatments, pathologic data, use of endocrine therapy (tamoxifen or aromatase inhibitors), and disease status at the time of last contact or death. The body weight and height values used were those obtained and recorded in the EMR by clinic staff at each routine oncology clinic visit.

For weight change analysis, baseline body weight was defined as weight within 90 days before and on the date closest to GLP1-RA initiation. Body weight was also collected at three time points after GLP1-RA initiation to evaluate relative change over time: 3 months (90 ± 45 days), 6 months (180 ± 45 days), and 12 months (360 ± 60 days). DFS was defined as the time from breast cancer diagnosis to disease recurrence or death due to breast cancer. OS was defined as the time from breast cancer diagnosis to death from any cause; patients alive at the time of analysis or lost to follow-up were censored at the last contact date.

### Statistical analysis

Continuous variables were summarized by mean, median, SD, range, and quartiles. Categorical variables were summarized using frequency tables. Weight change was summarized using box plots for patients who specifically received the agents semaglutide or tirzepatide (dual GLP1-RA/GIP-RA agonist); these GLP1-RAs, which are approved in the United States for weight loss, are now the most commonly used and most effective agents for this indication in contemporary practice. Statistical tests were performed using two-sided tests with 5% type I error rate. Univariate and multivariate linear regression models were used to assess the association of weight change with clinical factors (DM2, metformin use, endocrine therapy use, duration of GLP1-RA, menopausal status, and breast cancer stage) in GLP1-RA recipients with DCIS and invasive breast cancer. Estimates by regression coefficients (β) were provided.

For survival analysis, patients with DCIS were removed given their generally different disease trajectory and cancer outcomes compared with those with invasive disease. This left patients with stage I to III breast cancer and GLP1-RA nonusers. The GLP1-RA nonuser patients were identified and extracted from the MDACC BCMS database and included with those with stage I to III invasive breast cancer from 2005 to 2024 who did not receive any prior GLP1-RA (Fig. 1). Propensity score matching was used to match patients who received GLP1-RA with patients who did not (1:2 ratio, respectively) based on baseline clinical characteristics of BMI at breast cancer diagnosis (mean ± SD, kg/m^2^), DM2 (yes or no), age at breast cancer diagnosis (mean ± SD, years), cancer stage (I, II, or III), and breast cancer subtype [hormone receptor (HR) positive or HR negative]. Kaplan–Meier estimates and log-rank tests compared DFS and OS between treatment groups. Hazard ratios were estimated using Cox proportional hazards models. To address potential immortal time bias, we conducted landmark analyses at 6, 12, 24, 36, and 60 months after diagnosis (Supplementary Fig. S2), restricting the analysis to patients who survived and remained under observation at each respective time point. For each landmark, we recalculated OS starting from the landmark date and compared outcomes between the GLP-1RA users and GLP1-RA nonusers using Kaplan–Meier analysis and log-rank tests. All statistical analyses were performed using SAS version 9.4.

## Results

### Patient characteristics

Of a total of 48,768 patients with breast cancer in the database from years 2005 to 2024, the cohort of GLP1-RA recipients for at least 3 months with weight data available and with DCIS or stage I to III invasive breast cancer included 1,022 patients (Fig. 1; Table 1). The median follow-up time from breast cancer diagnosis was 4.7 years (range, 0–30.9 years). Patients were 99.3% female (*n* = 1,015) and predominantly over the age of 50 years (642, 62.8%); most patients (*n* = 581, 56.9%) were postmenopausal. There were 816 (79.8%) with DM2. A total of 653 (63.9%) received metformin and 487 (47.7%) received insulin.

**Table 1.** Baseline clinical characteristics of patients with breast cancer who received GLP1-RA.

Patients (*n* = 1,022)	*n* (%)
Sex	​
Female	1,015 (99.3%)
Male	7 (0.7%)
Age	​
Mean ± SD, years	54.07 ± 10.46
≤50 years	380 (37.2%)
>50 years	642 (62.8%)
Menopausal status	​
Postmenopausal	581 (56.8%)
Premenopausal	441 (43.2%)
Race/ethnicity	​
Non-Hispanic Black	181 (17.7%)
Non-Hispanic White	629 (61.5%)
Hispanic/Latino	150 (14.7%)
Other	62 (6.1%)
GLP1-RA type	​
Semaglutide	383 (37.5%)
Tirzepatide	59 (5.8%)
Lixisenatide	5 (0.5%)
Albiglutide	9 (0.9%)
Dulaglutide	310 (30.3%)
Exenatide	43 (4.2%)
Liraglutide	213 (20.8%)
DM2	816 (79.8%)
Metformin use	653 (63.9%)
Insulin use	487 (47.7%)
Breast cancer subtype	​
HR+/HER2+	80 (7.8%)
HR+/HER2−	624 (61.1%)
HR−/HER2+	50 (4.9%)
TNBC	142 (13.9%)
DCIS	125 (12.2%)
Unknown	1 (0.1%)
Cancer stage	​
DCIS	125 (12.2%)
I	376 (36.8%)
II	370 (36.2%)
III	144 (14.1%)
Receipt of chemotherapy	546 (53.4%)
Endocrine therapy use	673 (65.9%)
Tamoxifen	416 (40.7%)
Aromatase inhibitor	257 (25.1%)

The most common breast cancer subtype was HR positive/HER2 negative (624, 61.1%), followed by triple-negative breast cancer (TNBC; *n* = 142, 13.9%), DCIS (*n* = 125, 12.2%), HR positive/HER2 positive (*n* = 80, 7.8%), and HR negative/HER2 positive (*n* = 50, 4.9%). Clinical cancer stage distribution showed 36.8% (*n* = 376) stage I, 36.2% stage II (*n* = 370), 14.1% (*n* = 144) stage III, and 12.2% (*n* = 125) with DCIS. More than half (*n* = 546, 53.4%) received chemotherapy. Moreover, 65.9% (*n* = 673) received endocrine therapy. Specifically, 416 patients (40.7%) received tamoxifen and 257 (*n* = 25.2%) received aromatase inhibitors. Of those who received endocrine therapy, 38 (5.6%) received it overlapping (±90 days) with GLP1-RA.

### GLP1-RA characteristics

Of 1,022 patients, 989 (96.8%) received GLP-RA exclusively after breast cancer (no prescription prior to breast cancer diagnosis). The GLP1-RA types were semaglutide (*n* = 383, 37.5%), dulaglutide (*n* = 310, 30.3%), liraglutide (*n* = 213, 20.8%), tirzepatide (*n* = 59, 5.8%), exenatide (43, 4.2%), and lixisenatide and albiglutide in less than 1% each. GLP1-RA was started after definitive breast cancer therapy (chemotherapy, surgery, and radiation) in 83% (*n* = 848); the median time from breast cancer diagnosis to GLP1-RA initiation in these patients was 4.7 (range, 0–34) years. The median duration of GLP1-RA use was 1.2 (range, 0.3–8.1) years.

### Baseline weight and weight loss

**Figure 2.**
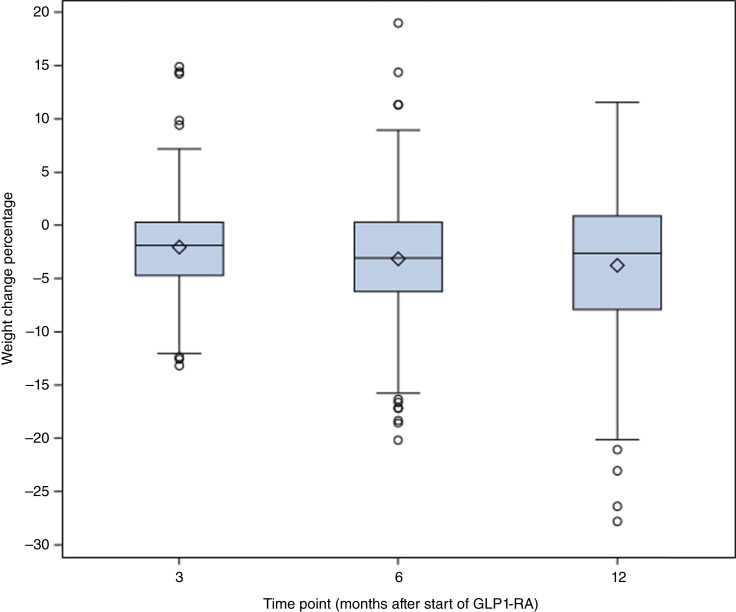
Box plot of weight change percentage at 3, 6, and 12 months in patients with DCIS and invasive breast cancer who received semaglutide or tirzepatide (*n* = 442, 43.2%). Shown are medians, IQRs, and ranges.

In all 1,022 patients who received GLP1-RA, the median BMI at breast cancer diagnosis was 33.3 kg/m^2^ (range, 20.2–56 kg/m^2^). Baseline median weight and median BMI at GLP1-RA initiation (within 90 days prior) were 86.8 kg (range, 47.2–175 kg) and 33.5 kg/m^2^ (range, 18.9–61.8 kg/m^2^), respectively. In patients who specifically received semaglutide or tirzepatide (*n* = 442, 43.2%), weight data were available in 22.2%, 23.4%, and 18.2% at 3, 6, and 12 months, respectively. The median weight change (range) at 3, 6, and 12 months in these patients was −1.9% (−13.2% to 14.9%), −3.1% (−20.2% to 19%), and −2.6% (−27.8% to 11.5%) from baseline weight, respectively (Fig. 2). In a subgroup of patients who did not have DM2 (*n* = 206), the median weight change (range) at 3, 6, and 12 months was −1.3% (−13.2% to 9.9%), −2.3% (−18.6% to 19%), and −2.3% (−27.8% to 11.5%), respectively. In a subgroup of patients who were premenopausal (*n* = 441), the median weight change (range) at 3, 6, and 12 months was −2.9% (−13.2% to 14.9), −3.3% (−18.6% to 19.9), and −3.5% (−26.4% to 11.5%), respectively. In a subgroup of patients who were postmenopausal (*n* = 581), the median weight change (range) at 3, 6, and 12 months was −1.8% (−12.6% to 14.4%), −2.9% (−20.2% to 11.3%), and −2.4% (−27.8% to 11.6%), respectively.

In an exploratory analysis of patients who received semaglutide or tirzepatide, we also evaluated the 6- and 12-month median weight change in the subgroup of patients who received the GLP1-RA prescription for at least 6 (*n* = 175) and 12 (*n* = 133) months, respectively, as well as predictors of weight change in these subgroups (Supplementary Tables S1 and S2).

### Predictors of weight change

The 3-month percentage change in weight was significantly associated with metformin use in both univariate (*P* = 0.011) and multivariate (*P* = 0.007) regression analysis (Table 2). Specifically, in multivariate analysis, patients who received metformin experienced 1.9% greater weight loss on average at 3 months compared with those who did not receive metformin. However, there was no significant association between weight change at 3 months and DM2, endocrine therapy use, duration of GLP1-RA, menopausal status, or breast cancer stage in univariate or multivariate analysis. There was no significant association observed between weight change at 6 months and any clinical factors in both univariate and multivariate analysis.

**Table 2.** Univariate and multivariate regression analysis of predictors of weight change from baseline at 3, 6, and 12 months.

Variable	Category	Estimate (β)	*P* value
Univariate regression analysis at 3 months			
Stage	Invasive disease vs. DCIS	−1.22	0.251
Endocrine therapy	Yes vs. No	−0.68	0.272
GLP1-RA use duration (year)		−0.02	0.944
DM2	Yes vs. No	−0.18	0.790
Metformin use	Yes vs. No	−1.57	**0.011**
Menopausal status	Post vs. Pre	0.30	0.634
Multivariate regression analysis at 3 months			
Stage	Invasive disease vs. DCIS	−1.29	0.236
Endocrine therapy	Yes vs. No	−0.60	0.338
GLP1-RA use duration (year)		0.17	0.590
DM2	Yes vs. No	0.65	0.406
Metformin use	Yes vs. No	−1.88	**0.007**
Menopausal status	Post vs. Pre	0.30	0.645
Univariate regression analysis at 6 months			
Stage	Invasive disease vs. DCIS	0.72	0.619
Endocrine therapy	Yes vs. No	−0.28	0.734
GLP1-RA use duration		−0.05	0.896
DM2	Yes vs. No	−0.42	0.632
Metformin use	Yes vs. No	−0.21	0.796
Menopausal status	Post vs. Pre	−0.13	0.875
Multivariate regression analysis at 6 months			
Stage	Invasive disease vs. DCIS	0.73	0.613
Endocrine therapy	Yes vs. No	−0.24	0.780
GLP1-RA use duration (year)		0.005	0.990
DM2	Yes vs. No	−0.21	0.840
Metformin use	Yes vs. No	−0.11	0.908
Menopausal status	Post vs. Pre	−0.01	0.991
Univariate regression analysis at 12 months			
Stage	Invasive disease vs. DCIS	−4.02	**0.015**
Endocrine therapy	Yes vs. No	2.36	**0.035**
GLP1-RA use duration (year)		0.426	0.454
DM2	Yes vs. No	−0.68	0.573
Metformin use	Yes vs. No	1.60	0.138
Menopausal status	Post vs. Pre	0.58	0.593
Multivariate regression analysis at 12 months			
Stage	Invasive disease vs. DCIS	−4.31	**0.010**
Endocrine therapy	Yes vs. No	1.93	0.088
GLP1-RA use duration (year)		0.33	0.568
DM2	Yes vs. No	−2.11	0.115
Metformin use	Yes vs. No	1.76	0.136
Menopausal status	Post vs. Pre	0.97	0.384

*P* values that are bolded were statistically significant.

Weight change at 12 months was significantly associated with endocrine therapy in univariate analysis, in which tamoxifen or aromatase inhibitor use was associated with weight gain (*P* = 0.035). Patients who received endocrine therapy experienced, on average, a 2.4% greater weight gain at 12 months compared with those who did not. However, in multivariate analysis, endocrine therapy was no longer significantly associated with weight change (*P* = 0.088). Similarly, weight change at 12 months was significantly associated with stage, such that those with invasive breast cancer lost more weight than those with DCIS in both univariate (*P* = 0.015) and multivariate (*P* = 0.010) analysis. Specifically, in multivariate analysis, patients with invasive breast cancer experienced 4.3% greater weight loss at 12 months compared with those with DCIS. There was no significant association between weight change at 12 months and other clinical factors (univariate or multivariate analysis).

### Survival outcome analysis

For the propensity-matched survival analysis of patients with invasive breast cancer, 810 patients receiving GLP1-RA were matched with 1,620 patients who did not receive GLP1-RA (Table 3). Although the raw means for BMI and diabetes status (DM2) seem to be different in Table 3, we assessed covariate balance using standardized mean differences (SMD), a more appropriate metric for balance in matched data (16). All covariates had SMDs <0.1 after matching, including BMI (SMD = 0.10) and DM2 (SMD = −0.01), indicating good balance. We have included a Love plot (Supplementary Fig. S1) to visually display covariate balance before and after propensity matching.

**Table 3.** Baseline clinical characteristics of GLP1-RA user and nonuser cohorts in propensity matching analysis^a^.

Patients	Patients prescribed GLP1-RA (*N* = 810)	Patients not prescribed GLP1-RA (*N* = 1,620)
BMI^a^ (mean ± SD, kg/m^2^)	34.21 ± 6.84	31.41 ± 6.80
Sex	​	​
Female	805 (99.4%)	1,602 (98.9%)
Male	5 (0.6%)	18 (1.1%)
Age^a^ (mean ± SD, years)	54.24 ± 10.52	56.04 ± 11.65
Menopausal Status	​	​
Postmenopausal	469 (57.9%)	1,323 (81.7%)
Premenopausal	341 (42.1%)	297 (18.3%)
Race/ethnicity	​	​
Non-Hispanic Black	143 (17.7%)	273 (16.9%)
Non-Hispanic White	506 (62.5%)	1,049 (64.8%)
Hispanic/Latino	124 (15.3%)	0
Other	37 (4.6%)	267 (16.5%)
Unknown	NA	31 (1.9%)
DM2^a^	642 (79.3%)	1,059 (65.4%)
Metformin use	517 (63.8%)	731 (45.1%)
Insulin use	394 (48.6%)	562 (34.7%)
Breast cancer subtype^a^	​	​
HR+	645 (79.6%)	443 (27.3%)
HR−	165 (20.4%)	1,177 (72.7%)
Cancer stage^a^	​	​
I	343 (42.4%)	620 (38.3%)
II	338 (41.7%)	697 (43%)
III	129 (15.9%)	303 (18.7%)
Receipt of chemotherapy	490 (60.5%)	1,056 (65.2%)
Endocrine therapy use	557 (68.8%)	1,028 (63.4%)
Tamoxifen	193 (23.8%)	355 (21.9%)
Aromatase inhibitor	364 (44.9%)	673 (41.5%)

a Matching factors included BMI at breast cancer diagnosis, DM2 diagnosis, age, breast cancer stage, and breast cancer subtype.

**Figure 3.**
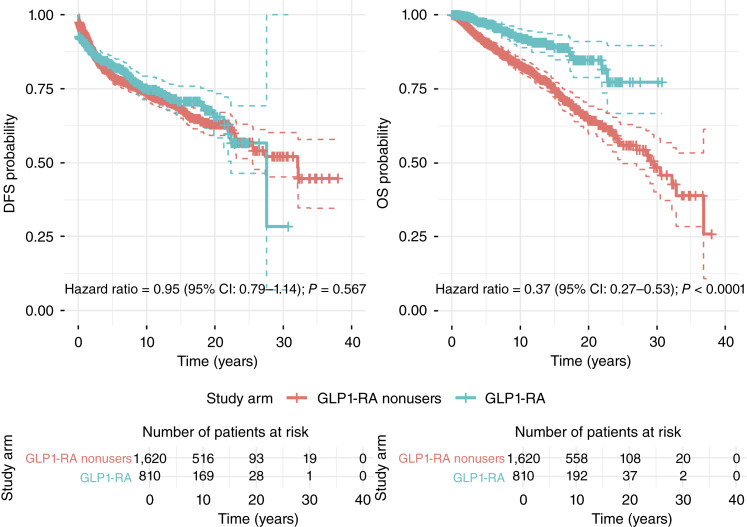
Kaplan–Meier estimates of DFS and OS in patients with invasive breast cancer who received and did not receive GLP1-RA.

DFS did not significantly differ between GLP1-RA users and nonusers (Fig. 3), with a median of 27.5 [95% confidence interval (CI), 21.92–not reached and range, 0–30.7] years in the GLP1-RA group versus 32 (95% CI, 25.49–not reached and range, 0–38) years in the GLP1-RA nonuser group (hazard ratio, 0.95; 95% CI, 0.79–1.14; *P* = 0.567). However, OS was significantly improved in the GLP1-RA group (Fig. 3), with median survival not reached (range, 0–30.7) in the GLP1-RA group versus 29.5 (95% CI, 24.63–36.84 and range, 0–38) years in the GLP1-RA nonuser group (hazard ratio, 0.37; 95% CI, 0.27–0.53; *P* < 0.0001). Our results also consistently demonstrated significantly improved OS in the GLP-1RA group across all landmark points, with log-rank *P* values < 0.001 at 6, 12, 24, 36, and 60 months (Supplementary Fig. S2).

## Discussion

To our knowledge, this the largest study to date describing GLP1-RA use in patients with breast cancer. In this population, we shed light on real-world patterns of weight loss and clinical outcomes (recurrence risk and survival) associated with these widely used pharmaceuticals approved for DM2 and weight loss. A sample of more than 1,000 patients with breast cancer who received any GLP1-RA within a single institution highlights the uptake of these drugs in a modern cohort of adult cancer survivors. Moreover, although the majority (79.8%, *n* = 816) had a DM2 diagnosis, 20.2% (*n* = 206) notably did not. This latter group presumably received GLP1-RA for weight loss, representing the largest report of breast cancer survivors who received this class of drugs for a weight management indication alone.

We observed successful weight loss at 3, 6, and 12 months from GLP1-RA in both those with and without DM2 who received semaglutide or tirzepatide, the agents most clinically utilized for weight management. However, the median weight change of only −3.1% at 6 months and −2.6% at 12 months was lower than expected, particularly when considering the magnitude observed in the general adult population, in which approximately 10% to 15% weight loss is expected at a similar time point from these agents (17, 18). Our findings of only modest weight loss are consistent with three other single-institution retrospective studies in patients with breast cancer. In one abstract of patients with HR-positive breast cancer who received endocrine therapy, at 12 months after GLP1-RA initiation, a mean weight change of −4.34% (95% CI, −7.7% to −1%) was observed with semaglutide and −2.31% (95% CI, −11.45% to 20.5%) with tirzepatide (13). In another study of 75 patients with breast cancer (85% with stage I–III disease and 83% with an HR-positive subtype), the median weight change at 12 months after GLP1-RA initiation was −4.2 kg (95% CI, −2.1 to −6.2 kg), or approximately 5%, and there was no association between covariates (baseline BMI, concurrent endocrine therapy, duration of GLP1-RA, and DM2 diagnosis) and weight change (19). Similarly, in an abstract by Fischbach and colleagues (15) of 70 patients with stage I to III invasive breast cancer treated with semaglutide or tirzepatide, the mean weight loss was 3 kg (95% CI, 2.1–3.9 kg), and the mean maximal weight loss was 8.9 kg (95% CI, 6.7–11 kg) over the GLP1-RA duration. In summary, although direct comparisons between studies are not feasible, the signal of attenuated weight loss effect observed among multiple separate breast cancer cohorts is perplexing and warrants further investigation.

One potential explanation for reduced weight loss compared with a non–breast cancer population and existing clinical trial data may be due to variations in drug dosing, schedule, and treatment adherence. Additionally, this may relate to endocrine therapy given that antiestrogen treatments in women with HR-positive breast cancer are linked to weight gain (20, 21). Approximately two thirds of our study population received endocrine therapy, and we found that its use was significantly associated with decreased weight loss efficacy at 12 months. Patients who received prior endocrine therapy contradictorily experienced, on average, weight gain despite receipt of GLP1-RA. However, in multivariate regression, controlling for other factors, the use of endocrine therapy no longer significantly affected relative weight change. Emerging evidence suggests synergy between the estrogen and GLP1 receptor, and future studies should explore the body composition effect from concurrent GLP1-RA and endocrine therapy use (22). Given the small sample of those in our dataset receiving both GLP1-RA and endocrine therapy at overlapping time points (*n* = 38), this analysis was not feasible. Moreover, the clinical implications when GLP1-RA is administered with other classes of anticancer therapies are not well elucidated. This is particularly relevant in patients undergoing treatment with agents contributing to metabolic dysfunction (e.g., chemotherapy, PI3K/AKT/mTOR inhibitors, and immune checkpoint inhibitor therapy; refs. 14, 23, 24).

With regard to the relationship of other clinical factors and weight loss, metformin use was associated with modestly increased weight loss with GLP1-RA at 3 months but not at 6 or 12 months. Metformin is associated with favorable metabolic effects, including modest weight loss in some individuals (25). Moreover, cancer stage was significantly associated with weight loss at 12 months as those with invasive breast cancer experienced more weight loss with GLP1-RA compared with patients with DCIS. This result could be related to the differential uptake of endocrine therapy in DCIS versus invasive disease; however, further study is warranted to better understand this association.

After propensity matching using multiple prognostic factors, we did not identify a DFS benefit with GLP1-RA compared with those who did not receive the GLP1-RA. These findings are similar to those of another study that evaluated breast cancer outcomes when GLP1-RA was administered after a breast cancer diagnosis that did not find a difference in locoregional or distant breast cancer recurrence in those who received semaglutide or tirzepatide compared with those who did not (15). Our findings are also consistent with existing epidemiologic data in cancer prevention research (26, 27). Namely, a study utilizing the national TriNetX platform to review more than 1.6 million patients with DM2 who received GLP1-RA evaluated the incident risk of developing multiple cancers (26). Compared with insulin, GLP1-RA use was associated with a decreased risk of developing 10 obesity-related malignancies (26); however, GLP1-RA use was not associated with reduced risk of postmenopausal breast cancer (26). Similarly, in a meta-analysis of more than 50 randomized clinical trials (women aged 45–70 years and 7.5-year follow-up), there was no significant difference in breast cancer in patients who received GLP1-RA compared with placebo or other antidiabetic agents (28). Another population-based cohort study (nearly 45,000 women 40 years and older and mean follow-up of 3.5 years) also showed no difference in the risk of breast cancer development with the use of GLP1-RA compared with other antidiabetic drugs (29).

These observational data in the pre– and post–breast cancer diagnosis setting suggesting no impact on breast cancer outcomes conflict with preclinical findings in which GLP1-RA has favorable antineoplastic effects (bioRxiv 2024.01.20.576484; refs. 30, 31). In one study of a murine model with obesity-driven endometrial cancer, tirzepatide inhibited both body weight and tumor growth through direct effects on immune and metabolic pathways of the tumor (31). This included modulation in genes (e.g., downregulation of ErbB signaling pathway and increase in B-cell receptor signaling) and mediation of glycolysis/fatty acid metabolism (31). In another study with a TNBC mouse model of diet-induced obesity, tirzepatide resulted in sustained weight loss and suppressed mammary tumor growth (bioRxiv 2024.01.20.576484). Notably, chronic caloric restriction promoted even greater weight loss and tumor suppression (bioRxiv 2024.01.20.576484). Clinical trials and prospective studies are crucial to shed light on potential anticancer effects of GLP1-RA and whether such findings could be related to a direct antiproliferative biologic impact versus a more indirect effect (i.e., related to favorable metabolic regulation as a result of caloric restriction and weight loss). Moreover, the optimal timing to introduce these pharmaceuticals in the context of the cancer care continuum is unanswered.

The improved all-cause mortality in GLP1-RA users despite no observed difference in breast cancer outcomes is intriguing. These findings remained consistent after landmark analyses at several time points after breast cancer diagnoses, reinforcing the robustness of our results and indicating that the survival benefit observed is unlikely to be solely driven by immortal time bias. This survival benefit could potentially be explained by the established benefits of GLP1-RA for health outcomes. Beyond their known efficacy for glycemic control and weight loss, GLP1-RA can mitigate risk related to broad pleiotropic effects on multiple organ systems in adult populations. Examples include reduced cardiovascular disease risk and renal protective effects. Emerging data now extend to benefits in other comorbid conditions, such as substance use/addiction and steatotic liver disease (9–12, 32). Moreover, the association of GLP1-RA with improved OS in patients with DM2 is well established (33, 34).

This retrospective cohort study has limitations. A single-center study could limit generalizability of the results and future investigation should encompass diverse breast cancer populations across representative geographic and sociodemographic groups. Although we adjusted for multiple prespecified factors, there remains the possibility of residual confounding. While we matched multiple prognostic factors in the outcome analysis, we could not account for all differences (e.g., socioeconomic status), which could have inadvertently affected results, and there is also the potential for selection bias affecting survival results. This is important given that socioeconomic status may contribute to disparities in access to these therapies, owing to their high cost, and may likewise affect breast cancer survival. Moreover, a median follow-up time of 4.7 years is relatively short, considering the natural history of breast cancer. Additionally, because of limitations in prescription data within the database, we were unable to delineate the precise drug exposure with each GLP1-RA to account for certain factors related to dosing and schedule (e.g., medication adherence and treatment interruptions), which could have affected weight loss data and the outcome assessment. GLP1-RA exposure was defined based on EMR-documented prescriptions and pharmacy dispensing dates, and continued use was inferred from prescription refills and absence of documented noncompliance in follow-up clinic visits. Although this approach captures prescribing and likely patient use, it does not allow direct confirmation of adherence. Misclassification of GLP1-RA use is a potential limitation as some exposures may not have been captured in the electronic health record. However, most GLP1-RA use in this cohort occurred before the widespread off-label or nonprescription use for weight management became common, reducing the likelihood of significant under-ascertainment. Still, unmeasured use (e.g., via compounding pharmacies) may have occurred and could bias the observed associations. Future studies should entail more granular data in this regard and also separate DM2 versus non-DM2 diagnoses because of differences in drug prescriptions between these indications.

### Conclusions

In a cohort of more than 1,000 breast cancer survivors, GLP1-RA use was associated with modest weight loss; however, endocrine therapy could have decreased this impact. Metformin use and cancer stage may also affect weight loss. An improvement in all-cause survival was observed but not a significant difference in breast cancer recurrence risk. Clinical trials are needed to investigate the role of these agents for weight loss as an adjunct to lifestyle interventions in patients with breast cancer with obesity, including those with and without DM2. Further exploration of their long-term safety and potential anticancer biological effects, particularly in the context of breast cancer risk reduction and cancer control, is also warranted.

## Supplementary Material

Supplementary Figure 1 Figure S1. Love Plot: Standardized Mean Differences Before and After Propensity Score Matching

Supplementary Figure 2 Figure S2. Landmark Analysis of Overall Survival at 6, 12, 24, and 36, and 60 months after breast cancer diagnosis

Supplementary Tables 1 and 2 Table S1. Weight change at 6 and 12 months in the patients who received semaglutide or tirzepatide for at least 6 months (n=175) and at least 12 months (n=133), respectively. Table S2. Univariate and Multivariate Regression Analysis of predictors of weight change from baseline at 6 and 12 months in the patients who received semaglutide or tirzepatide for at least 6 and 12 months, respectively.

## Data Availability

The data that support the findings of this study are not publicly available because of patient privacy concerns; however, they are available upon reasonable request from the corresponding author.

## References

[1] Siegel RL , Giaquinto AN, Jemal A. Cancer statistics, 2024. CA Cancer J Clin 2024;74:12–49. 38230766 10.3322/caac.21820

[2] Shelby RA , Dorfman CS, Arthur SS, Bosworth HB, Corsino L, Sutton L, . Improving health engagement and lifestyle management for breast cancer survivors with diabetes. Contemp Clin Trials 2020;92:105998. 32289471 10.1016/j.cct.2020.105998 PMC7590108

[3] Chan DS , Vieira AR, Aune D, Bandera EV, Greenwood DC, McTiernan A, . Body mass index and survival in women with breast cancer-systematic literature review and meta-analysis of 82 follow-up studies. Ann Oncol 2014;25:1901–14. 24769692 10.1093/annonc/mdu042 PMC4176449

[4] Sheng JY , Sharma D, Jerome G, Santa-Maria CA. Obese breast cancer patients and survivors: management considerations. Oncology (Williston Park) 2018;32:410–7. 30153321 PMC9337744

[5] Feigelson HS , Bodelon C, Powers JD, Curtis RE, Buist DSM, Veiga LHS, . Body mass index and risk of second cancer among women with breast cancer. J Natl Cancer Inst 2021;113:1156–60. 33823007 10.1093/jnci/djab053 PMC8757319

[6] Runowicz CD , Leach CR, Henry NL, Henry KS, Mackey HT, Cowens-Alvarado RL, . American Cancer Society/American Society of Clinical Oncology Breast Cancer Survivorship Care Guideline. J Clin Oncol 2016;34:611–35. 26644543 10.1200/JCO.2015.64.3809

[7] Seino Y , Fukushima M, Yabe D. GIP and GLP-1, the two incretin hormones: similarities and differences. J Diabetes Investig 2010;1:8–23. 10.1111/j.2040-1124.2010.00022.x PMC4020673 24843404

[8] Nauck MA , Quast DR, Wefers J, Pfeiffer AFH. The evolving story of incretins (GIP and GLP-1) in metabolic and cardiovascular disease: a pathophysiological update. Diabetes Obes Metab 2021;23(Suppl 3):5–29. 34310013 10.1111/dom.14496

[9] Xie Y , Choi T, Al-Aly Z. Mapping the effectiveness and risks of GLP-1 receptor agonists. Nat Med 2025;31:951–62. 39833406 10.1038/s41591-024-03412-w

[10] Kristensen SL , Rørth R, Jhund PS, Docherty KF, Sattar N, Preiss D, . Cardiovascular, mortality, and kidney outcomes with GLP-1 receptor agonists in patients with type 2 diabetes: a systematic review and meta-analysis of cardiovascular outcome trials. Lancet Diabetes Endocrinol 2019;7:776–85. 31422062 10.1016/S2213-8587(19)30249-9

[11] Lincoff AM , Brown-Frandsen K, Colhoun HM, Deanfield J, Emerson SS, Esbjerg S, . Semaglutide and cardiovascular outcomes in obesity without diabetes. N Engl J Med 2023;389:2221–32. 37952131 10.1056/NEJMoa2307563

[12] Chuang M-H , Chen J-Y, Wang H-Y, Jiang Z-H, Wu V-C. Clinical outcomes of tirzepatide or GLP-1 receptor agonists in individuals with type 2 diabetes. JAMA Netw Open 2024;7:e2427258. 39133485 10.1001/jamanetworkopen.2024.27258 PMC11320168

[13] Urueta Portillo D , Mazo-Canola M, Alhaj S. Evaluating the impact of GLP-1 receptor agonists on weight management in patients with breast cancer undergoing endocrine therapy: a comparative analysis. J Clin Oncol 2024;42(Suppl 16):e13063.

[14] Shen S , Chen Y, Carpio A, Chang C, Iyengar NM. Incidence, risk factors, and management of alpelisib-associated hyperglycemia in metastatic breast cancer. Cancer 2023;129:3854–61. 37743730 10.1002/cncr.34928 PMC10863751

[15] Fischbach N , Zhou B, Deng Y, Parsons K, Shelton A, Lustberg MB. Impact of semaglutide and tirzepatide administration on weight in women with stage I-III breast cancer. J Clin Oncol 2024;42(Suppl 16):e24140.

[16] Austin PC . Balance diagnostics for comparing the distribution of baseline covariates between treatment groups in propensity-score matched samples. Stat Med 2009;28:3083–107. 19757444 10.1002/sim.3697 PMC3472075

[17] Jastreboff AM , Aronne LJ, Ahmad NN, Wharton S, Connery L, Alves B, . Tirzepatide once weekly for the treatment of obesity. N Engl J Med 2022;387:205–16. 35658024 10.1056/NEJMoa2206038

[18] Wilding JPH , Batterham RL, Calanna S, Davies M, Van Gaal LF, Lingvay I, . Once-weekly semaglutide in adults with overweight or obesity. N Engl J Med 2021;384:989–1002. 33567185 10.1056/NEJMoa2032183

[19] Shen S , Liu B, Fanti C, Bromberg M, Chen Y, Chang C, . GLP-1 receptor agonist use and weight change in patients with breast cancer. Oncology (Williston Park) 2025;7:294–6. 10.46883/2025.25921046 40834286

[20] Raghavendra A , Sinha AK, Valle-Goffin J, Shen Y, Tripathy D, Barcenas CH. Determinants of weight gain during adjuvant endocrine therapy and association of such weight gain with recurrence in long-term breast cancer survivors. Clin Breast Cancer 2018;18:e7–13. 29239836 10.1016/j.clbc.2017.11.006 PMC5937690

[21] Uhelski A-CR , Blackford AL, Sheng JY, Snyder C, Lehman J, Visvanathan K, . Factors associated with weight gain in pre- and post-menopausal women receiving adjuvant endocrine therapy for breast cancer. J Cancer Surviv 2024;18:1683–96. 37261654 10.1007/s11764-023-01408-y PMC11424737

[22] Tiano JP , Tate CR, Yang BS, DiMarchi R, Mauvais-Jarvis F. Effect of targeted estrogen delivery using glucagon-like peptide-1 on insulin secretion, insulin sensitivity and glucose homeostasis. Sci Rep 2015;5:10211. 25970118 10.1038/srep10211 PMC4429560

[23] Durán JGB , Das D, Gildea M, Amadori L, Gourvest M, Kaur R, . Immune checkpoint landscape of human atherosclerosis and influence of cardiometabolic factors. Nat Cardiovasc Res 2024;3:1482–502. 39613875 10.1038/s44161-024-00563-4 PMC11634783

[24] Dieli-Conwright CM , Wong L, Waliany S, Bernstein L, Salehian B, Mortimer JE. An observational study to examine changes in metabolic syndrome components in patients with breast cancer receiving neoadjuvant or adjuvant chemotherapy. Cancer 2016;122:2646–53. 27219902 10.1002/cncr.30104 PMC4992442

[25] Yerevanian A , Soukas AA. Metformin: mechanisms in human obesity and weight loss. Curr Obes Rep 2019;8:156–64. 30874963 10.1007/s13679-019-00335-3 PMC6520185

[26] Wang L , Xu R, Kaelber DC, Berger NA. Glucagon-like peptide 1 receptor agonists and 13 obesity-associated cancers in patients with type 2 diabetes. JAMA Netw Open 2024;7:e2421305. 38967919 10.1001/jamanetworkopen.2024.21305 PMC11227080

[27] Wang L , Wang W, Kaelber DC, Xu R, Berger NA. GLP-1 receptor agonists and colorectal cancer risk in drug-naive patients with type 2 diabetes, with and without overweight/obesity. JAMA Oncol 2024;10:256–8. 38060218 10.1001/jamaoncol.2023.5573 PMC10704339

[28] Piccoli GF , Mesquita LA, Stein C, Aziz M, Zoldan M, Degobi NAH, . Do GLP-1 receptor agonists increase the risk of breast cancer? A systematic review and meta-analysis. J Clin Endocrinol Metab 2021;106:912–21. 33248445 10.1210/clinem/dgaa891

[29] Hicks BM , Yin H, Yu OHY, Pollak MN, Platt RW, Azoulay L. Glucagon-like peptide-1 analogues and risk of breast cancer in women with type 2 diabetes: population based cohort study using the UK Clinical Practice Research Datalink. BMJ 2016;355:i5340. 27797785 10.1136/bmj.i5340

[30] Fidan-Yaylalı G , Dodurga Y, Seçme M, Elmas L. Antidiabetic exendin-4 activates apoptotic pathway and inhibits growth of breast cancer cells. Tumour Biol 2016;37:2647–53. 26399993 10.1007/s13277-015-4104-9

[31] Kong W , Deng B, Shen X, John C, Haag J, Sinha N, . Tirzepatide as an innovative treatment strategy in a pre-clinical model of obesity-driven endometrial cancer. Gynecol Oncol 2024;191:116–23. 39388742 10.1016/j.ygyno.2024.10.004 PMC12419173

[32] Nevola R , Epifani R, Imbriani S, Tortorella G, Aprea C, Galiero R, . GLP-1 receptor agonists in non-alcoholic fatty liver disease: current evidence and future perspectives. Int J Mol Sci 2023;24:1703. 36675217 10.3390/ijms24021703 PMC9865319

[33] Chen J-J , Wu C-Y, Jenq C-C, Lee T-H, Tsai C-Y, Tu H-T, . Association of glucagon-like peptide-1 receptor agonist vs dipeptidyl peptidase-4 inhibitor use with mortality among patients with type 2 diabetes and advanced chronic kidney disease. JAMA Netw Open 2022;5:e221169. 35254430 10.1001/jamanetworkopen.2022.1169 PMC8902651

[34] Guler G , Himmetoglu C, Jimenez RE, Geyer SM, Wang WP, Costinean S, . Aberrant expression of DNA damage response proteins is associated with breast cancer subtype and clinical features. Breast Cancer Res Treat 2011;129:421–32. 21069451 10.1007/s10549-010-1248-6 PMC3677189

