# Supplementary figures and images for: Weight Loss Patterns and Clinical Outcomes of GLP1 Receptor Agonists in Breast Cancer Survivors
Source: Cancer Res Commun. 2026 Mar 2;6(3):447–55. doi: 10.1158/2767-9764.CRC-25-0554 (PMC13043164; doi:10.1158/2767-9764.CRC-25-0554)

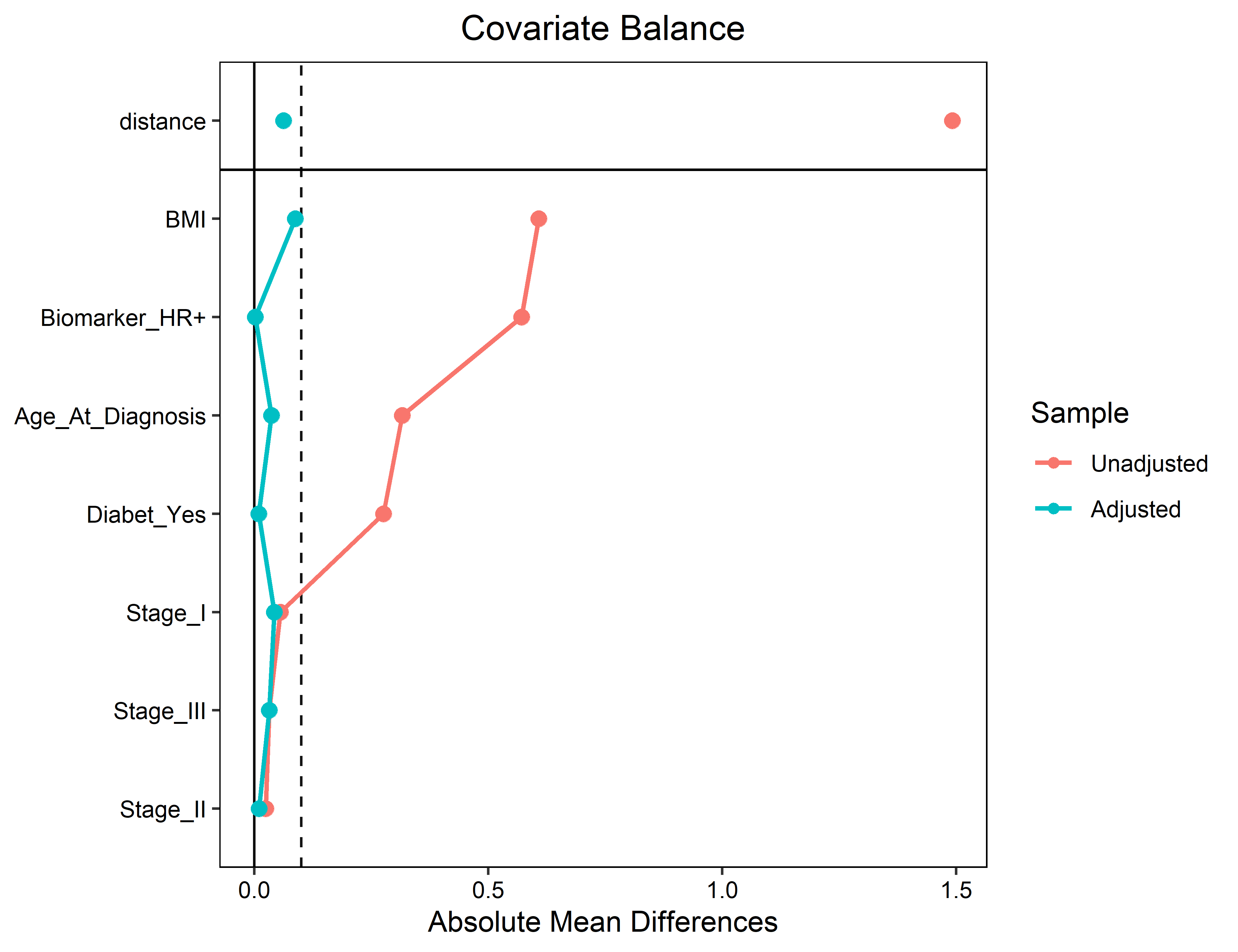

Supplement: Supplementary Figure 1 — Figure S1. Love Plot: Standardized Mean Differences Before and After Propensity Score Matching [file crc-25-0554_supplementary_figure_1_suppsf1.png]

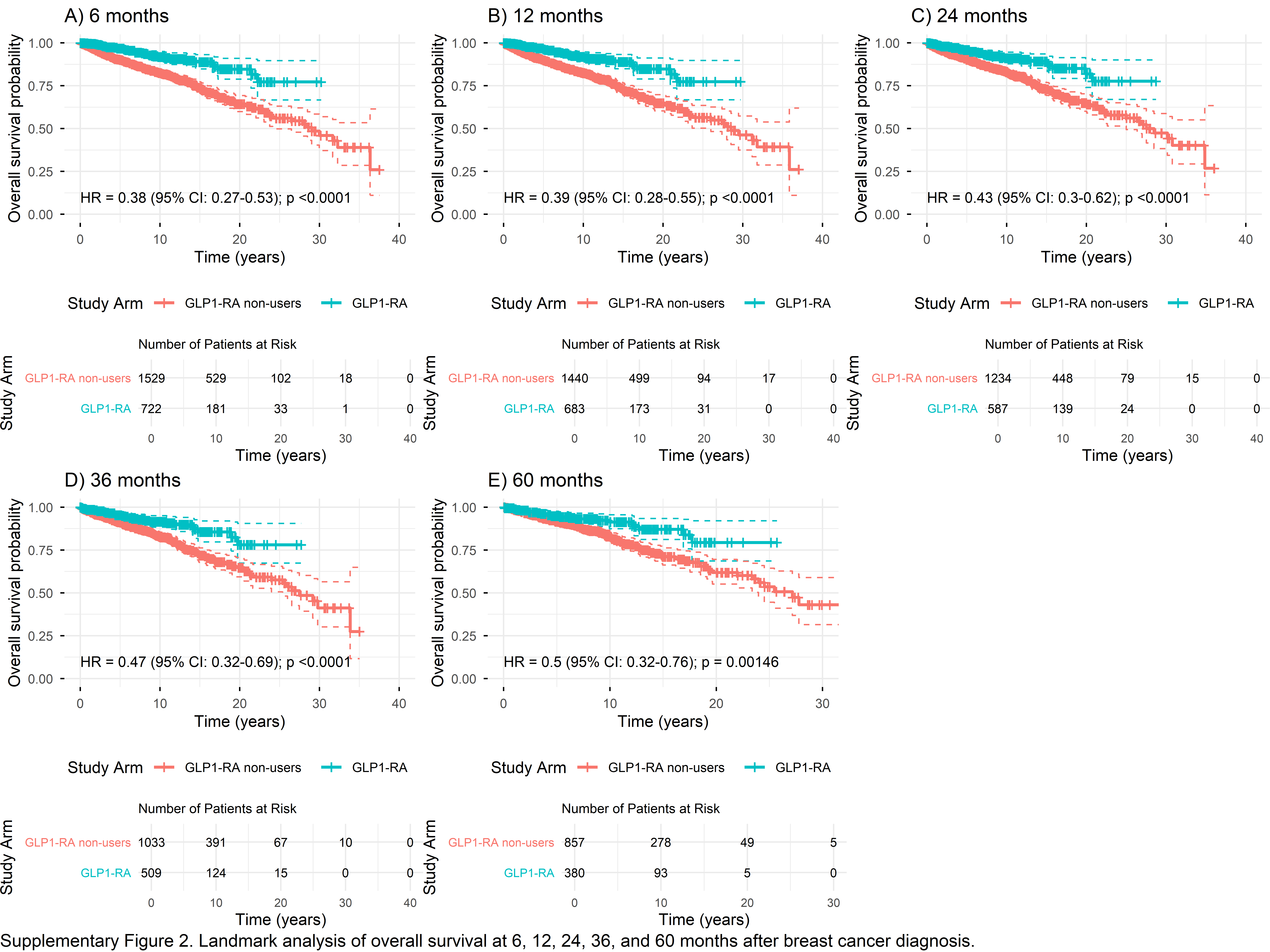

Supplement: Supplementary Figure 2 — Figure S2. Landmark Analysis of Overall Survival at 6, 12, 24, and 36, and 60 months after breast cancer diagnosis [file crc-25-0554_supplementary_figure_2_suppsf2.png]
