# Supplementary material for: Weight Loss Patterns and Clinical Outcomes of GLP1 Receptor Agonists in Breast Cancer Survivors
Source: Cancer Res Commun. 2026 Mar 2;6(3):447–55. doi: 10.1158/2767-9764.CRC-25-0554 (PMC13043164; doi:10.1158/2767-9764.CRC-25-0554)
Supplement: Supplementary Tables 1 and 2 — Table S1. Weight change at 6 and 12 months in the patients who received semaglutide or tirzepatide for at least 6 months (n=175) and at least 12 months (n=133), respectively. Table S2. Univariate and Multivariate Regression Analysis of predictors of weight change from baseline at 6 and 12 months in the patients who received semaglutide or tirzepatide for at least 6 and 12 months, respectively. [file crc-25-0554_supplementary_tables_1_and_2_suppst1.docx]

**Supplementary Table 1:** Weight change at 6 and 12 months in the patients who received semaglutide or tirzepatide for at least 6 months (n=175) and at least 12 months (n=133), respectively

| **Variable** | **Median (range)** |
| --- | --- |
| % Weight change at 6 months | -3.3 (-20.2 - 14.4) |
| % Weight change at 12 months | -2.7 (-27.8 - 11.5) |

**Supplementary Table 2:** Univariate and Multivariate Regression Analysis of predictors of weight change from baseline at 6 and 12 months in the patients who received semaglutide or tirzepatide for at least 6 and 12 months, respectively.

| **Univariate regression analysis at 6 months** | | | |
| --- | --- | --- | --- |
| **Variable** | **Category** | **Estimate (β)** | **p-value** |
| Stage | Invasive disease vs DCIS | -0.13 | 0.925 |
| Endocrine therapy | Yes vs No | 0.57 | 0.483 |
| GLP1-RA use duration | | 0.41 | 0.296 |
| Diabetes type 2 | Yes vs No | 0.80 | 0.369 |
| Metformin use | Yes vs No | 0.54 | 0.498 |
| Menopausal status | Post vs Pre | 0.99 | 0.220 |
| **Multivariate regression analysis at 6 months** | | | |
| Stage | Invasive disease vs DCIS | -0.40 | 0.786 |
| Endocrine therapy | Yes vs No | 0.44 | 0.605 |
| GLP1-RA use duration (year) | | 0.24 | 0.564 |
| Diabetes type 2 | Yes vs No | 0.39 | 0.705 |
| Metformin use | Yes vs No | 0.08 | 0.931 |
| Menopausal status | Post vs Pre | 0.90 | 0.302 |
| **Univariate regression analysis at 12 months** | | | |
| Stage | Invasive disease vs DCIS | -3.24 | 0.053 |
| Endocrine therapy | Yes vs No | 2.07 | 0.092 |
| GLP1-RA use duration (year) | | 0.56 | 0.355 |
| Diabetes type 2 | Yes vs No | -1.77 | 0.171 |
| Metformin use | Yes vs No | 1.16 | 0.322 |
| Menopausal status | Post vs Pre | 0.89 | 0.439 |
| **Multivariate regression analysis at 12 months** | | | |
| Stage | Invasive disease vs DCIS | -3.79 | **0.023** |
| Endocrine therapy | Yes vs No | 1.67 | 0.170 |
| GLP1-RA use duration (year) | | 0.75 | 0.73 |
| Diabetes type 2 | Yes vs No | -3.55 | **0.014** |
| Metformin use | Yes vs No | 1.85 | 0.139 |
| Menopausal status | Post vs Pre | 1.27 | 0.277 |
